# Early handling and repeated cross-fostering have opposite effect on mouse emotionality

**DOI:** 10.3389/fnbeh.2015.00093

**Published:** 2015-04-21

**Authors:** Alessandra Luchetti, Diego Oddi, Valentina Lampis, Eleonora Centofante, Armando Felsani, Marco Battaglia, Francesca R. D’Amato

**Affiliations:** ^1^Institute of Cell Biology and Neurobiology, National Research Council/Fondazione Santa LuciaRome, Italy; ^2^Academic Centre for the Study of Behavioral Plasticity, Vita-Salute San Raffaele UniversityMilan, Italy; ^3^GenomniaLainate, Italy; ^4^Institut Universitaire en Santé Mentale de Québec, Laval UniversityQuebec, QC, Canada

**Keywords:** early adversities, mice, maternal behavior, HPA axis, respiratory response to hypercapnia, panic disorder, attachment behavior

## Abstract

Early life events have a crucial role in programming the individual phenotype and exposure to traumatic experiences during infancy can increase later risk for a variety of neuropsychiatric conditions, including mood and anxiety disorders. Animal models of postnatal stress have been developed in rodents to explore molecular mechanisms responsible for the observed short and long lasting neurobiological effects of such manipulations. The main aim of this study was to compare the behavioral and hormonal phenotype of young and adult animals exposed to different postnatal treatments. Outbred mice were exposed to (i) the classical Handling protocol (H: 15 min-day of separation from the mother from day 1 to 14 of life) or to (ii) a Repeated Cross-Fostering protocol (RCF: adoption of litters from day 1 to 4 of life by different dams). Handled mice received more maternal care in infancy and showed the already described reduced emotionality at adulthood. Repeated cross fostered animals did not differ for maternal care received, but showed enhanced sensitivity to separation from the mother in infancy and altered respiratory response to 6% CO_2_ in breathing air in comparison with controls. Abnormal respiratory responses to hypercapnia are commonly found among humans with panic disorders (PD), and point to RCF-induced instability of the early environment as a valid developmental model for PD. The comparisons between short- and long-term effects of postnatal handling vs. RCF indicate that different types of early adversities are associated with different behavioral profiles, and evoke psychopathologies that can be distinguished according to the neurobiological systems disrupted by early-life manipulations.

## Introduction

The developmental programming hypothesis suggests that the early environment, whether by nutritional, hormonal or behavioral processes, can give rise to persistent modifications of the adult phenotype. In particular, when facing a challenging environment, epigenetic modifications may occur that modify the behavioral, physiological, hormonal and neurobiological profile of the developing individual, to optimize its future coping strategies (Bock et al., 2014). Several studies in rodents have investigated the effects of a challenging environment, experimentally altering the external or internal pup’s milieu, and various postnatal manipulations, differing for severity, time and duration schedules have been applied to developing animals. In the majority of studies (but see also Moles et al., 2004b, 2008), pups were directly stressed exposing them to low temperature, poor mothering, saline injection, unfamiliar odors and others (Oddi et al., 2015). The most common manipulation applied to developing rodents consisted in exposing young animals to daily sessions of separation from the mother during the first 1–2 weeks of life (Pryce and Feldon, 2003). Maternal separation is adversative and pups search for the mother by emitting calls and by seeking olfactory and thermal cues of her presence. This indicates the establishment of an attachment bond between the infant and the mother in the first 2 weeks of life, with signs of distress (e.g., ultrasonic vocalizations (USVs)) following maternal separation that are already detectable in the first few postnatal days (PND0). According to the duration of the stress sessions and the age of pups, different aversive experiences were recruited: pups experienced body temperature loss, starvation, absence of familiar and presence of new odors, absence or excess of tactile stimulation and so on. Moreover, according to the duration of separation sessions, different effects have been observed in the mother and, during development and at adulthood, in the offspring. The hypothalamus-pituitary-adrenal (HPA) axis functioning is greatly affected by these early separation sessions, with opposite behavioral and hormonal responses to stress observed in adult age, according to the duration of the separation events (Anisman et al., 1998; Nishi et al., 2013; but see also Faturi et al., 2010). In many studies pups, repeatedly subjected to long separations from the mother, showed depression- and anxiety-like behaviors in adulthood (Newport et al., 2002; Daniels et al., 2004; Lee et al., 2007; Ryu et al., 2009).

Rather than repeated separations, unpredictability of the early environment may represent a stressful condition for pups. Repeated cross-fostering (RCF) has been used in mice as a postnatal manipulation to model human early environmental instability, a risk factor for internalizing disorders (including separation anxiety disorder—SAD-, panic disorder—PD- and CO_2_ hypersensitivity, Kendler et al., 1992; Forman and Davies, 2003; Battaglia et al., 2009). Even though animal models are not expected to reproduce clinical disorders exactly, a translational model of PD should allow to differentiate panic attack (PA) from fear, on the basis of respiratory symptoms (over-reaction to hypercapnia) and lack of increments in stress hormones (Schenberg et al., 2014). Cross-fostering is a routine procedure used in many laboratories that consists in giving pups to a lactating female different from the biological mother, usually within 24–48 h from birth (Oddi et al., 2015). RCF consists in repeating the same procedure every day for the first 4 days of life. Changes in maternal (olfactory, gustatory, tactile, thermal, etc.) cues connected with the RCF procedure may disrupt the associative learning process that is necessary for establishment of the attachment bond in the developing infant (Landers and Sullivan, 2012).

The temporary separation from the mother, or the absence/malfunctioning of the attachment bond (RCF protocol) may act on different molecular system and differently affect the development of emotionality and vulnerability to specific psychopathologies. For example, it is known that separation protocols in rodents strongly affect the HPA axis, whereas corticosterone baseline levels are not affected by the RCF protocol, at least in young animals (D’Amato et al., 2011). Consistent with these data, depression and anxiety disorders, as modeled by separation protocols, are usually associated with alterations in HPA axis functioning, whereas PD, modeled by RCF, is not, at least during the first phases of illness (Klein, 1993).

Here, we evaluated the short- and long-term behavioral effects of two different manipulations of the early environment. In one case pups experienced short separations (Handling) from the mother, which interferes with continuity of the bond; in the other case, pups experienced the Repeated Cross-Fostering procedure, which is aimed at interfering with bond formation. Some data suggest that handled pups should show reduced behavioral and hormonal response to stress at adulthood (Meaney et al., 1996), whereas RCF mice should develop deficit in the attachment and reward system, together with hyper-responsiveness to CO_2_ in inhaled air (Oddi et al., 2015). However, the effects of maternal separation in rodents-mice especially-yield little agreement among laboratories and strains (see for example Millstein and Holmes, 2007).

To help resolving these issues, we analyzed the specificities of the RCF vs. Handling protocols effects on behavioral readouts and on the panic-related respiratory responses to CO_2_ among outbred strains in the same laboratory. Different response to these manipulations would support the relative selectivity of behavioral and molecular mechanisms involved in response to different types of adversities.

## Methods

### Animals

NMRI outbred mice (Harlan, Italy) were used in all experiments. Mice were mated when they were 12 weeks old. Mating protocol consisted in housing 2 females with 1 male in transparent high temperature polysufone cages (26.7 × 20.7 × 14.0 cm) with water and food available *ad libitum*. Room temperature (21 ± 1°C) and a 12:12 h light dark cycle (lights on at 07.00 p.m.) were kept constant. After 15 days, males were removed and pregnant females were isolated, left in clean cages, and inspected twice a day for live pups.

All animal used procedures were in strict accordance with standard ethical guidelines (European Community Guidelines on the Care and Use of Laboratory Animals 86/609/EEC) and the Italian legislation on animal experimentation (Decreto L.vo 116/92).

### Experimental Manipulations

On PND1 litters were culled to 8 pups (4 males and 4 females) and assigned to handling (H) or repeated cross-fostering (RCF) procedure.

#### Handling

According to the well-validated paradigm called “handling” (e.g., Pryce et al., 2005), pups were briefly handled and separated from the dam for 15 min daily. This procedure took place from PND1 to PND14 between 9:30 and 11:00 am. Control litters (N-H), once completed the culling procedure, were left undisturbed for the first 2 weeks of life.

#### Repeated Cross-Fostering

**Figure 1:**
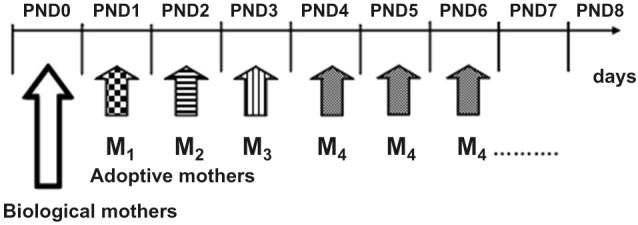
**Schematic representation or the Repeated Cross-Fostering (RCF) protocol**. Pups changed caregiver every 24 h, from birth (PND0) till PND4 experiencing a total of five different “mothers” (1 biological plus 4 adoptive mothers).

After having spent the first postnatal day (PND0) with the biological mother, on PND1 culled litters were assigned to experimental Repeated Cross Fostering or control (N-RCF) treatment. Differently from the “classical” cross-fostering procedures (Bartolomucci et al., 2004), RCF pups changed caregiver every 24 h: 4 times in the PND1-PND4 time interval by following a rotation scheme, each dam shifted to 4 different litters and each litter was shifted to 4 different dams (see also Figure 1). The daily procedure consisted of first removing the mother from the cage, then removing its entire litter, and immediately introducing this litter into the home-cage of a different dam whose pups had just been removed. The RCF pups were then semi-covered with the home-cage bedding of the adoptive mother, which was then reintroduced in the cage and left with this litter for the next 24 h. The entire procedure lasted about 30 s and took place every day between 10.30 and 11.00 a.m. This was repeated daily, four times until reaching the fourth adoptive mother, with which pups were left until weaning (PND0: biological mother, PND1-PND4: adoptive mother 1–4, PND4-PND28: fourth adoptive mother- Figure 1). Adoptive dams were lactating females with pups of the same age as fostered litters. Control litters (N-RCF) were picked up daily and reintroduced in their home-cage, covered with home-cage bedding and had their biological mothers returned within 30 s; this procedure took place from PND1 to PND4 in order to control the possible effect of manipulation necessarily required by RCF procedure.

A total of four experimental groups resulted from the early manipulations: handled and their controls (H and N-H), RCF and their controls (RCF and N-RCF). Animals were weaned when 28 days old, and then separated by sex and left in cage with littermates. Only male mice were tested from weaning onwards.

### Short and Long-Term Effects of Postnatal Manipulations

The effects of H and RCF on offspring were compared according to eight different physiological, molecular, and behavioral parameters collected during development and adulthood. Body weight (1) was measured in infancy (PND8) and adulthood (PND90). Maternal behavior (2) was observed during the first week of life to exclude the action of poor nurturing on offspring’s responses. USVs (3) in response to isolation (PND8), and sociability and social preference (4) were measured before (PND28) and after weaning (PND35), respectively. Adult males (PND75–90) were also tested for behavioral emotionality (5), HPA functionality as indicated by corticosterone response to stress (6) and hippocampal mRNA levels of the glucocorticoid and mineralocorticoid receptors (7). In addition, respiratory responses (8) to a 6% CO_2_-enriched air mixture were evaluated in adult animals. No more than 2 animals per litter were tested on the same task.

#### Behavioral Measures on Infant-Mother Bond

##### Maternal Behavior

Maternal behavior was observed daily from PND2 to PND7 by an observer unaware of the litter’s manipulation (H, N-H, RCF and N-RCF) in two daily sessions (12.00–12.30 and 16.00–16.30) in the facility room. The first daily session took place at least 1 h after the cross fostering/maternal separation procedures, in order to facilitate the dams’ acclimatization. Maternal behavior encompassing: (a) NURSING, including the arched-back and blanket postures; and (b) GP/L: grooming and licking pups was monitored with an instantaneous sampling method (1 sample every 2 min), for a total of 16 sampling points/session (Shoji and Kato, 2006). The analyzes of maternal behaviors were based on the observation of NURSING and GP/L on 15 litters of RCF, 16 litters of N-RCF, 10 litters of H and 11 litters of N-H pups.

Data were analyzed by two way ANOVAs, the factors being (1) manipulation (4 levels: H, N-H, RCF and N-RCF); and (2) developmental age (2 levels repeated measure: PND2–4 and PND 5–7). The observation period was split into 2 time-windows: PND2–4 (daily cross—fostering period) and PND5–7 (definitive adoption for the RCF group) to control for the immediate effect of the RCF protocol.

##### Ultrasonic Vocalizations

Pups’ behavior was evaluated at PND8, by measuring USVs emitted during 5 min of isolation (Moles et al., 2004a; Cryan and Holmes, 2005). Experimental animals were transferred in their home cage to the experimental rooms for USVs assessment, 1 h prior to testing. After this period of acclimatization, the mother was removed and transferred into a clean cage, while pups were left in the home cage standing on a warm plate set at the temperature of 35°C to prevent cooling. One randomly chosen pup was placed into a beaker, containing own-cage bedding and the vocalizations were recorded. No more than 1 pup/litter was employed. USVs were recorded using an UltraSoundGate Condenser Microphone (CM16, Avisoft Bioacoustics, Berlin, Germany) lowered 1 cm above the top of the isolation beaker containing the pup. The microphone was sensitive to frequencies of 15–180 kHz with a flat frequency response (± 6 dB) between 25–140 kHz. It was connected via a UltraSoundGate USB Audio device to a personal computer, where acoustic data were recorded as wav files at 250,000 Hz in 16 bit format. Sound files were transferred to SasLab Pro (version 4.40; Avisoft Bioacoutics) for sonographic analysis and a fast Fourier transformation was conducted (512 FFT-length, 100% frame, Hamming window and 75% time window overlap). Further details on this procedure, the device used and the analysis of data can be found in D’Amato et al. (2011).

A one-way ANOVA, the factor being manipulation (4 levels: H, N-H, RCF and N-RCF), was used to compare the total number of vocalizations emitted by pups during the 5 min of isolation session. The sex of the pup was not considered as we never observed a male-female difference in 8-day old pups’ ultrasonic emission (D’Amato et al., 2011; Cinque et al., 2012).

#### Sociability and Social Preference

Sociability and social preferences were evaluated in male mice at PND28 (before weaning), and at PND35 (1 week after weaning), respectively, in different animals (Cinque et al., 2012). Measures of interest in an unknown conspecific vs. an unknown object were employed as indicators of sociability. Indices of social preference were acquired to test whether H and RCF affected siblings’ recognition. The social preference test was performed 1 week after weaning to reduce the impact of the mother on sibling’s olfactory cues. Both tests used a gray Plexiglas rectangular box (60 × 40 × 24 cm) consisting of three interconnected chambers. Each of the two lateral compartments contained a circular Plexiglas cylinder (diameter: 8 cm, height: 15 cm) with multiple holes (diameter: 1.2 cm) yielding olfactory cues. Mouse behavior was recorded by a video-camera and analyzed with the SMART video-tracking system. Each subject mouse was placed inside the central compartment and explored the apparatus for a 10-min habituation period, with the doors on either side left open. During the 10 min social session of the test, the tested animal was exposed to an unfamiliar animal and a white object of similar size (Sociability test), or was simultaneously exposed to an unfamiliar (same strain, age and treatment) and a familiar male mouse (sibling) (Social preference test). Each partner and object was confined in one of the two Plexiglas cylinder located in the lateral compartments, for 10 min. The position of stimuli (partners and objects) in the apparatus was equally distributed between the left and the right compartment. Collected measures included time spent: (a) in each one of the three compartment; and (b) in the immediate proximity (2 cm: Time Close) of each cylinders.

One-way ANOVAs, the factor being manipulation (4 levels: H, N-H, RCF and N-RCF), were conducted on a Sociability and Social Preference index that measured the percentage of time spent close to unfamiliar partners (Time Close unfamiliar/(Total Time close to both cylinders) × 100).

#### Emotionality

Male mice were tested in the elevated plus maze at PND75–90 for emotionality. No more than 2 males × litter for group were sampled. The elevated plus maze consisted of 2 open (5 cm wide, 30 cm long) and 2 closed arms (5 cm wide, 30 cm long, enclosed by a wall of 14 cm in height) arranged in a plus configuration, joined by a central square of 5 cm × 5 cm. The apparatus was made of opaque Plexiglas and kept on a base 40 cm above the floor. All animals were exposed to a test of a standard 5-min duration. At the beginning of the test each mouse was placed individually in the center of the maze, with the head facing an open-arm (the same for all mice). All tests were conducted between 13:00 h and 15:00 h and recorded by a video camera. The animals were initially accustomed to the experimental room for at least 1 h before the experiment.

The time spent in the different arms of the apparatus was evaluated by automatic software analysis (SMART, PanLab) and the percentage of time spent in open arms was used as behavioral index of low emotionality (100 × Time Open/(Time Open + Time Closed) in a one-way ANOVA, the factor being the postnatal manipulation (4 levels: H, N-H, RCF and N-RCF).

#### HPA Axis Functionality

##### Corticosterone response to novelty

Corticosterone levels were measured in H, N-H, RCF and N-RCF male mice, at different time intervals from novelty exposure. Apart from the postnatal manipulation, these animals have never been exposed to other experimental procedures. Novelty consisted in exposing the animals to a novel environment: each mouse was removed from its home cage and placed in the center of an open circular arena (60 cm diameter) for 20 min. Trunk blood samples were collected at different time intervals after the novelty test. One group of animals for each treatment was not manipulated at all and blood collected represented the group baseline (Time 0′). Immediately at the end of the novelty exposure, 50% of mice were sacrificed to measure the stress response to the open arena (Time 20′), while the other 50% was reintroduced in their home cages and blood was collected after 40 min (Time 60′). After blood centrifugation (20 min, 4°C, 4000 rpm), serum samples were stored at −25°C until assay were conducted. Corticosterone levels were measured using commercially available EIA kits (Enzo Life Science, sensitivity 27.0 pg/mL). All corticosterone measures were carried out in duplicate. The mean serum corticosterone levels of mice were compared by a two-way ANOVA, the factors being (1) manipulation (4 levels: H, N-H, RCF and N-RCF); and (2) time intervals (3 levels: time 0, 20′ and 60′).

#### Hippocampal mRNA Analyses

##### GR and MR expression (Real-time PCR analysis)

Brains of adult male mice of the Time 0 groups for corticosterone essays were rapidly removed and placed onto an ice-cooled metal plate. Hippocampi were dissected and samples were immediately frozen on dry ice and stored at −80°C. RNA was extracted from homogenized hippocampi (*N* = 5/7 for each experimental group) using a Total RNA purification kit (Norgen Biotek, Thorold, ON, Canada) following the instructions of manufacturer. RNA quantity was determined by absorbance at 260 nm using a NanoDrop UV-VIS spectrophotometer (Thermo Fisher Scientific, Wilmington, DE, USA).

RNA was reverse-transcribed with a High-Capacity cDNA Reverse Transcription Kit (Applied Biosystem, Paisley, UK) according to the manufacturer’s instructions. Equal amounts of cDNA were then subjected to real-time PCR analysis with an Applied Biosystems 7900HT thermal cycler, using the SensiMix SYBR Kit (Bioline, London, UK) and specific primers, each at a final concentration of 200 nM (Nr3c1: sense: CCTCCCAAACTCTGCCTGG, antisense AGCACAAAGGTAATTGTGCTGT; Nr3c2: sense CGGCTTCAGCTGACCTTTGA, antisense TGGCTCTTGAGGCCATCTTT; Actb: sense CAATGAGCTGCGTGTGGC, antisense GTACATGGCTGGGGTGTTGA). Each measurement was performed in quadrupli-cate and each experiment in triplicate. The expression data were normalized using the expression values of Actb gene. Amplification efficiency for each primer pair was determined by amplification of a linear standard curve (from 0.1 ng to 20 ng) of total cDNA as assessed by A260 spectrophotometry. Standard curves displayed good linearity and amplification efficiency for all primer pairs.

Expression data were presented, after normalization, as the fold-changes over the expression values of control samples (H vs. N-H and RCF vs. N-RCF). Independent *t*-tests between treated and control delta Cts (H vs. N-H and RCF vs. N-RCF) were used to evaluate significant differences in gene expression.

#### Respiratory Response to CO_2_ Enriched Environment

We evaluated the ventilatory responses to 6% air-CO_2_ concentration in adult H, N-H, RCF and N-RCF animals. Since the RCF procedure evokes enhanced respiratory responsiveness to hypercapnia, an endophenotype of human PD (D’Amato et al., 2011; Battaglia et al., 2014), we were interested in assessing to what extent H may alter CO_2_ sensitivity. We measured the changes in tidal volume (i.e., the volume of air displaced between normal inspiration and expiration, TV) during 6% CO_2_-enriched air breathing (CO_2_ challenge) in an unrestrained plethysmograph (PLY4211, Buxco Electronics, Sharon CT) carrying two separate Plexiglass chambers of 450 ml. This allows for the parallel assessment of 2 animals/session. Before any recording, each subject was closed in its chamber for an acclimatization of 40 min. Then, the recording of respiratory parameters started under air condition (baseline) for 20 min. Next, the challenge began with the administration of 6% CO_2_ enriched air, followed by a 20 min recovery period (air). A complete session thus lasted 80 min per animal. In a previous study (D’Amato et al., 2011) the complete procedure consisted in two subsequent challenges per animal with 6% CO_2_ enriched air, but since the correlation of the respiratory responses between the two challenges was found very high (>0.80), in the present study we relied on 1 challenge only.

A one way ANOVA, the factor being manipulation (4 levels: H, N-H, RCF and N-RCF), was used to compare the mean percentage of increment of tidal volume from baseline (ΔTV%) during 6% CO_2_ exposure (D’Amato et al., 2011).

## Results

### Body Weight

No effect of postnatal manipulation on body weight at PND8 and PND90 emerged (Table 1).

**Table 1:** **Mean body weight (gr.) of animals exposed to different postnatal manipulations, after USVs (PND8) and at adulthood, after respiratory parameters evaluation (PND90)**.

	PND8	PND90
H	6.52 + 0.08	45.70 + 1.52
N-H	6.67 + 0.29	49.98 + 2.09
RCF	6.57 + 0.10	50.20 + 1.13
N-RCF	6.23 + 0.15	46.24 + 2.21
One-way ANOVA	*F*_(3/23)_ = 1.27	*F*_(3/30)_ = 1.79
N	6–8	6–11

### Behavioral Measures on Infant-Mother Interactions

#### Maternal Behavior

**Figure 2:**
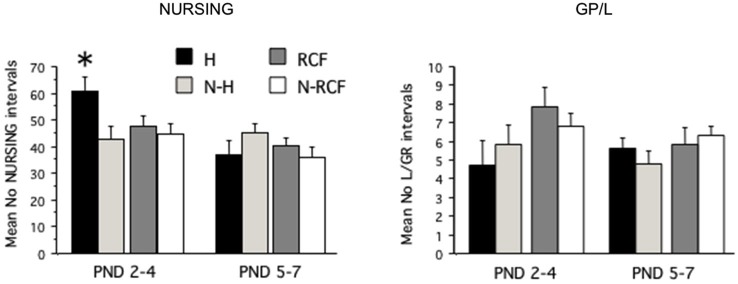
**Maternal care received by pups exposed to different postnatal manipulations**. Data are presented as mean (+SE) group scores for 3-day intervals (PND2–4 and PND5–7). Experimental groups: H: Handled (*n* = 10); N-H: Non-Handled (*n* = 11); RCF: Repeated Cross-Fostering (*n* = 15); N-RCF: Control (*n* = 16). *p < 0.05 in comparison with other groups of the same time interval (Fisher test).

The total amount of nursing and grooming behavior received by pups exposed to different manipulations is shown in Figure 2. The statistical analysis revealed that different manipulations did not affect the total amount of nursing and grooming/licking received by pups during the first week of life (NP: *F*_(3/48)_ = 1.00, ns; GP/L: *F*_(3/48)_ = 1.67, ns) but, while NURSING decreased during the first week of life (*F*_(1/48)_ = 14.27, *p* < 0.001), pups’ grooming and licking remained relatively stable (*F*_(1/48)_ = 1.41, ns) across all 4 experimental groups. The interaction between postnatal manipulation and time reached statistical significance only for NURSING (NP: *F*_(3/48)_ = 3.80, *p* < 0.02; GP/L: *F*_(3/48)_ = 0.98, ns). As is clearly depicted in Figure 2, H pups received more nursing than all other groups, but only during PND2–4. The amount of nurturing received by both control groups (N-H and N-RCF) was very similar.

#### Ultrasonic Vocalizations

**Figure 3:**
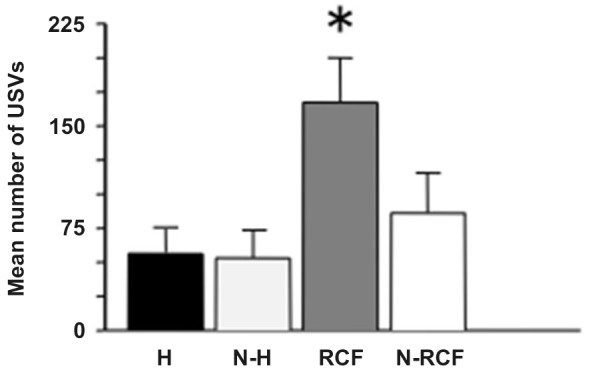
**Mean (+SE) number of ultrasonic calls (USVs) emitted by 8-day old pups of different experimental groups, when isolated in their own home-cage bedding for 5 min**. Sample sizes: 6–8 per group. Experimental groups: H: Handled; N-H: Non-Handled; RCF: Repeated Cross-Fostering; N-RCF: Control. *p < 0.05 in comparison with all other groups (Fisher test).

The response to isolation measured in pup on PND8 is shown in Figure 3: the ANOVA indicated a significant difference between groups (*F*_(3/23)_ = 4.30, *p* < 0.05). RCF pups emitted the highest number of USVs in comparison with all other groups during the 5 min session. Again, the 2 control groups (N-H and N-RCF) confirmed similar.

### Sociability and Social Preference

**Figure 4:**
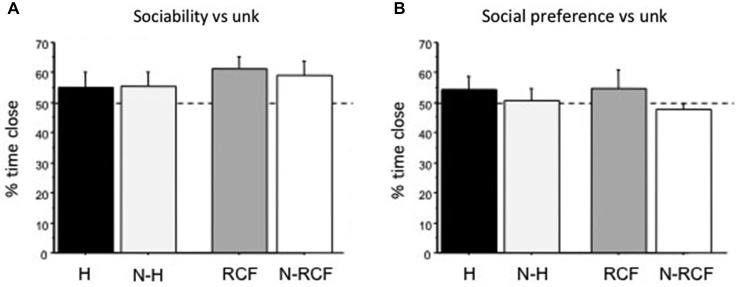
**Sociability and Social Preference scores (mean + SE). (A)** Sociability (preference for conspecific vs. object) and **(B)** Social preference (preference for conspecific vs. littermate) for unfamiliar male mouse (same strain, age and treatment) of juvenile males tested on PND28 and PND35, respectively. Both indices are calculated as the percentage of time spent close to unfamiliar partners (Time Close unfamiliar/(Total Time close to both cylinders) × 100). Sample sizes: 9–14 per group. Experimental groups: H: Handled; N-H: Non-Handled; RCF: Repeated Cross-Fostering; N-RCF: Control.

Young male mice explored the 3-compartment cage during the habituation sessions and no difference in the time spent in the different chambers was detected (data not shown, available from authors on request). Neither sociability towards unfamiliar partners (Figure 4A: *F*_(3/42)_ = 0.77, ns), nor social preference (Figure 4B: *F*_(3/47)_ = 1.22, ns) were affected by early manipulations. Considering time spent close to cylinders, more than 50% of this time involved exploration of the unfamiliar mouse and no preference/avoidance of siblings was detected.

### Emotionality

**Figure 5:**
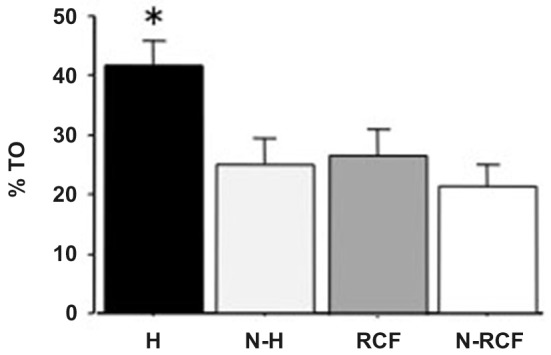
**Mean (+SE) percent of time spent in the open arms of an elevated plus maze by adult male mice exposed to different postnatal manipulations**. Sample sizes: 9–10 per group. Experimental groups: H: Handled; N-H: Non-Handled; RCF: Repeated Cross-Fostering; N-RCF: Control. *p < 0.05 in comparison with other experimental groups (Fisher test).

Postnatally-handled adult males showed, as expected, reduced emotionality in the plus maze test (Figure 5). The one-way ANOVA indicated a significant treatment effect (*F*_(3/33)_ = 4.43, *p* < 0.01) and *post-hoc* analyzes showed that the effect was explained by pups exposed to H manipulation.

### HPA Axis Functionality

#### Corticosterone Levels After Novelty Exposure

**Figure 6:**
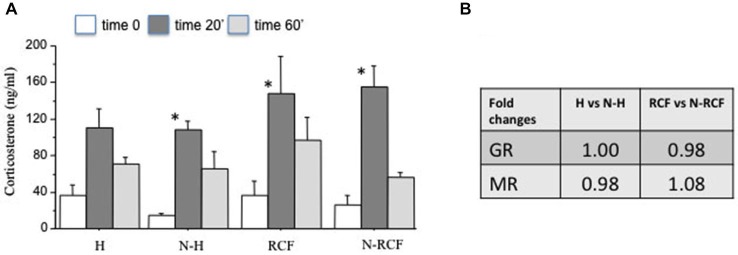
**HPA axis functionality. (A)** Mean (+SE) serum corticosterone levels of male mice from different experimental groups before (Time 0), at the end of novelty (Time 20′), and 40 min after reintroduction in their home cage (Timer 60′). Sample sizes: 6–7 per group. *p < 0.05 in comparison with same experimental group, different time interval. **(B)** Fold changes of hippocampal mRNA for Glucocorticoid (GR) and Mineralocorticoid (MR) receptors. Sample sizes: 5–7 per group. Experimental groups: H: Handled; N-H: Non-Handled; RCF: Repeated Cross-Fostering; N-RCF: Control.

The corticosterone response to a novel situation in the 4 experimental groups is depicted in Figure 6A. Mice did not differ for the amount of time spent in the central part of the arena (*F*_(3/45)_ = 1.72, ns) during novelty exposure. All groups showed an increase in serum corticosterone at the end of the novelty test (20 min of open field) and a successive reduction of hormone levels during the 40 min of recovery in the home cage. The two-way ANOVA for repeated measures indicated a significant time effect (*F*_(2/63)_ = 31.59, *p* < 0.001) and no experimental group (*F*_(3/63)_ = 1.54, ns), or group × time (*F*_(6/63)_ = 0.76, ns) effect. However, subsequent Tukey *post hoc* analysis revealed that the increase in corticosterone at the end of the open field exposure (baseline vs. Time 20′) was significantly higher in all groups but not in the group exposed to handling during postnatal life.

### Hippocampal mRNA Analyses

#### GR and MR Expression in the Hippocampus

The results of GR and MR gene expression in the hippocampal region, evaluated by real-time PCR, indicated no significant differences between groups, either for GR and MR gene expression (Figure 6B). Both GR and MR Delta CTs did not differ either between N and N-H (*t*_(8)_ = 0, and *t*_(8)_ = 0, ns, respectively), or between RCF and N-RCF (*t*_(12)_ = 0.28 and *t*_(12)_ = −0.79, ns, respectively).

### Respiratory Response to CO_2_ Enriched Environment

**Figure 7:**
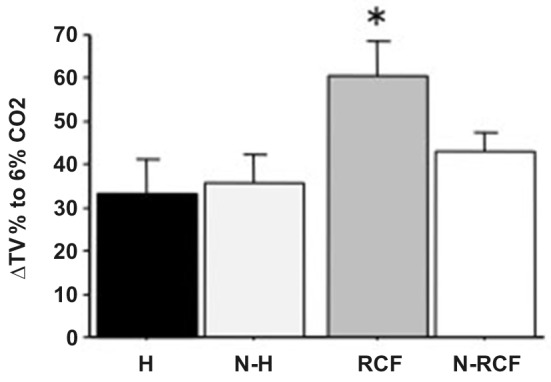
**Mean (+SE) percentage of Tidal Volume changes from baseline (ΔTV%) for adult male mice from different experimental groups, in response to 6% CO_2_**. Sample sizes: 6–11 per group. Experimental groups: H: Handled; N-H: Non-Handled; RCF: Repeated Cross-Fostering; N-RCF: Control. *p < 0.05 in comparison with other experimental groups (Fisher test).

Adult male mice responses to 6% CO_2_-enriched air are shown in Figure 7. The physiological increase in TV was significantly enhanced among RCF subjects (*F*_(3/30)_ = 3.64, *p* < 0.05) compared to all other groups.

## Discussion

The aim of this study was to compare the short (i.e., maternal behavior and pup’s body weight and USVs) and long lasting (i.e., sociability at weaning and adults’ emotionality, glucocorticoids mRNA and respiratory response to 6% CO_2_) neurobiological outcomes of two different postnatal manipulations, namely the H (whereby maternal separation is short and repeated) and the RCF (where maternal separation consists of repeated cross-fostering to lactating females other than the biological mother) protocols in NMRI outbred mice. Rodent studies of maternal separation and/or pup isolation in the first weeks of life were typically designed to approximate early-life adversities, to assess their psychobiological impact, and build proxies for human developmental psychopathology. The instability of the early environment induced by the RCF manipulation, represents an early aversive condition that has long-term effects, but does not to interfere with the HPA axis functioning. RCF pups were exposed to maternal cues from four different dams, a condition that might have prevented or disrupted infant-mother attachment bond. By studying rats, R. Sullivan found that the somatosensory, olfactory and gustatory stimulation associated with suckling, pups learned to recognize their mother (e.g., Sullivan et al., 2011). It is not clear whether the pups are able to recognize their mother or rather “a mother” as the presence of maternal signature odors (pheromone) has been reported for the rabbit and hypothesized in the mouse (Logan et al., 2012). We suggest that 8–10 day-old pups are able to recognize their own mother/nest environment, but the RCF protocol disrupts this preference. This deficit/malfunctioning of the attachment process, may simulate human separation events, that are risk factors for PD.

Results reported here are summarized in Table 2. Two points should be stressed before discussing results: (a) these studies were conducted on outbred mice a condition that can more closely be associated to the human situation; and (b) to facilitate comparisons with the previous, sometimes inconsistent, mouse studies of H (see for example Millstein and Holmes, 2007), and further extend knowledge on the recently-introduced RCF procedure (D’Amato et al., 2011; Ventura et al., 2013; Battaglia et al., 2014), in the table each manipulated group (H and RCF) was compared to the corresponding own control group (N-H and N-RCF animals).

**Table 2:** **Summary table reporting results of comparisons between each manipulated and its control group**.

Neurobiological/Behavioral indices	H vs. N-H	RCF vs. N-RCF
Maternal behavior received	>	ns
USVs response to isolation (m + f)	ns	>
Sociability (m)	ns	ns
Social preference (m)	ns	ns
Emotionality in the plus maze (m)	<	ns
Corticosterone response to novelty (m)	<	ns
Hippocampal GR and MR expression (m)	ns	ns
Respiratory response to CO_2_ adulthood (m)	ns	>
Body weight (m)	ns	ns

*H: Handled; N-H: Non Handled; RCF: Repeated Cross-Fostering; N-RCF: Controls for RCF; m: males; f: females*.

### Body Weight

First of all, data show that neither H nor RCF affected body weight during development or in adulthood. This suggests that neither manipulation induced generalized developmental perturbations.

### Maternal Cares, Emotionality and Glucocorticoids

Our results confirm that repeated short separation events (Handling) during the first 2 weeks of life promote heightened maternal care and are associated with reduced behavioral and hormonal reactivity to stress (plus maze and restraint stress) in adulthood, confirming data from many laboratories (e.g., Meaney et al., 1996; Schmidt et al., 2003). We were however not able to find, in our adult H mice, the increased expression of hippocampal GRs reported in the literature (Meaney et al., 1985; O’Donnell et al., 1994; Schmidt et al., 2003; George et al., 2013).

The RCF procedure, which implies a strong interference with the infant-mother attachment bond, yielded different short and long-term effects. Table 2 shows that RCF pups did not receive lower amount of maternal care compared to N-RCF, but responded to 5 min of isolation with a higher amount of distress calls. The fact that the RCF experimental procedure is based on the introduction of to-be-adopted pups into the adoptive dam’s home-cage covered with the adoptive mother’s bedding, may indeed explain the consistence of maternal cares across biological and adoptive mothers. No effect of RCF treatment on basal corticosterone levels in PND27 offspring and their mothers was found (D’Amato et al., 2011), in spite of the higher USV response to separation of RCF pups. This suggests that the RCF manipulation may hamper the pups’ ability to recognize the scent of their home cage and/or be reassured by being in an olfactory known-environment (D’Amato et al., 2011).

Contrary to handling, RCF protocol did not modify emotionality (plus maze) and hormonal response (corticosterone levels) to stress (Table 1). These results are not surprizing considering that differences in emotionality occurring in H adult animals have been explained by the different levels of maternal care received by these animals that would induce, through epigenetic response, changes in brain and behavior that persist until adulthood (Champagne et al., 2003). RCF pups received similar amount of care than controls and were never separated or isolated from a lactating female as H pups did, supporting absence of differences in emotionality between these two groups of animals. In addition H pups were exposed to fluctuation in corticosterone levels in the absence of the mother during the handling procedure, as well as to mother’s corticosterone levels changes that concomitantly occurred (D’Amato et al., 1992; Moles et al., 2008). By contrast, RCF pups and mothers were never separated and probably not exposed to such hormonal variations in early life.

### Social Preference

To reduce the impact of sexual hormones on sociability and social preferences parameters, we tested mice at weaning. Since both H and RCF may interfere with attachment, they may impact on social behavior (social preference/recognition of siblings) later in development. This was not the case, and neither H, nor RCF treatment affected social behaviors in young male mice. These animals are all interested in conspecifics and the postnatal treatment seems not to affect social motivation in immature mice.

### Sensitivity to Carbon Dioxide

As already reported in our previous study, RCF animals showed higher, stable and specific augmentation of tidal volume in response to 6% CO_2_-enriched air (D’Amato et al., 2011). This was confirmed here, as it was not seen among H animals, and unrelated to body weight between experimental groups. This hypersensitivity to CO_2_ can be turned into a remarkable investigational tool and useful endophenotype, allowing to model PD in the mouse. Animal models of PA in rats and mice are usually based on behavioral observations in classical anxiety tests, using pharmacological treatment (lactate) or electrical stimulation of the dorsal periaqueductal gray (dPAG) to exaggerate the response (e.g., Johnson and Shekhar, 2012; Andrews et al., 2014; Canteras and Graeff, 2014). Panic and anxiety disorders are usually discernible in humans on the basis of psychological and physical feelings, respiratory responses, and panic in the absence of real dangers that are largely independent of HPA axis activation (Abelson et al., 2007). The altered respiratory response to CO_2_ that characterized PD patients, as well as unaffected relatives, might represent a shared endophenotype that allow to investigate, in the mouse, the gene × environment interplay, the molecular mechanisms and functional alterations that characterize the psychopathology. The experience of early adversities that in humans influence—in addition to genetic factors-the risk for PD in adulthood, seem to be convincingly modeled by the RCF protocol in the mouse.

A methodological point is worth mentioning concerning the use of repeated cross-fostering as an early aversive event. Cross-fostering is a routine procedure in many laboratories and consists in giving pups to a lactating female different from the biological mother, usually within 24–48 h from birth (Oddi et al., 2015). This experimental protocol is used in several studies to improve maternal cares, to separate the effects of prenatal from postnatal treatments, to evaluate the effects of different amount of maternal care on offspring behavioral and physiological development, and to ameliorate sanitary condition in animal house by removing colony infections (see for example Barros et al., 2006; Buxbaum et al., 2011; Schmauss et al., 2014; Wattez et al., 2014). Few studies have investigated the effects of this procedure *per se* on later development. However, earlier adoptions exert smaller impacts than later ones, stressing that changes in mother’s cues are perceived by neonates and older may probably better detect differences than jounger pups, (Barbazanges et al., 1996; Hickman and Swan, 2011). These studies indicate that even if essential cares are provided by adoptive mothers, by substituting the biological mother in rodents one may affect body weight, emotional behavior and nociception in the offspring, and induce consistent metabolic and cardiovascular changes (Bartolomucci et al., 2004; Malkesman et al., 2008; Dickinson et al., 2009; Leussis and Heinrichs, 2009; Lu et al., 2009; Matthews et al., 2011).

## Conclusions

Generally speaking, results reported in this study, comparing in the same laboratory and in the same outbred mouse strain the short and long term effects of two different early treatments, suggest that the observed phenotypes depend on characteristics and timings of early adversities that might activate different biological processes. Reasonably, the response of the animal to the early manipulations is different and aimed at maximizing individual fitness: the early environment could exert its programming role during this developmental plastic period through specific epigenetic modifications. Short, even if repeated, separations from the mother (Handling protocol) induce habituation to a relatively low stressing environment, enhancing the capability of the subject to face new stressful situations. By contrast, the disruption of the infant attachment bond (RCF protocol) induces a modification in the respiratory response to high CO_2_ in breathing air, an endophenotype these animals shared with PD patients. The molecular mechanisms responsible for this increased response to acidosis in RCF mice are under investigation, as well as the impact of this early adversity on different genetic background, using genetic inbred lines of mice. This information will provide additional validity to this animal model of panic that shares etiology and respiratory endophenotype with the human PD.

## Conflict of Interest Statement

The authors declare that the research was conducted in the absence of any commercial or financial relationships that could be construed as a potential conflict of interest.
